# Linear accelerator-based stereotactic fractionated photon radiotherapy as an eye-conserving treatment for uveal melanoma

**DOI:** 10.1186/s13014-018-1088-9

**Published:** 2018-08-02

**Authors:** Sati Akbaba, Robert Foerster, Nils Henrik Nicolay, Nathalie Arians, Tilman Bostel, Juergen Debus, Henrik Hauswald

**Affiliations:** 1 Department of Radiation Oncology, University Hospital Heidelberg, Im Neuenheimer Feld 400, 69120 Heidelberg, Germany; 2 National Center for Radiation Research in Oncology (NCRO), Heidelberg Institute for Radiation Oncology (HIRO), Heidelberg, Germany; 3 Department of Radiation Oncology, University Hospital Zurich, Raemistrasse 100, 8091 Zurich, Switzerland; 4 Clinical Cooperation Unit Radiation Oncology, German Cancer Research Center (DKFZ), Im Neuenheimer Feld 280, 69120 Heidelberg, Germany

**Keywords:** Eye preservation, Organ preservation, Rare cancer, Sight preservation, Stereotactic radiotherapy, Toxicity, Uveal melanoma

## Abstract

**Background:**

The purpose of this retrospective analysis is to analyze clinical outcome, visual acuity and enucleation rates after linear accelerator-based stereotactic fractionated photon radiotherapy for primary uveal melanoma.

**Methods:**

Twenty-four patients with primary uveal melanoma treated at the Department of Radiation and Oncology of the University Hospital Heidelberg between 1991 and 2015 were analyzed regarding survival and treatment-related toxicity including eye- and sight-preservation.

**Results:**

Photon radiotherapy (RT) offered good overall local control rates with a local progression-free survival (LPFS) of 82% after 5 years and a median LPFS of 5.5 years at a median follow-up time of 5.2 years. Gender had a significant impact on LPFS yielding a mean LPFS of 8.1 years for women and 8.7 years for men (*p = 0.04*). Of all local progressions, 80% occurred within the first 5 years after RT. In one case, enucleation as final therapy option was necessary. Enucleation-free survival (EFS) was related to the radiotherapy dose (*p < 0.0001*). Thus, higher prescribed doses led to a significantly higher enucleation rate. T-stage had no significant impact on EFS, but affected the enucleation rate (*p = 0.01*). The overall survival (OS) rate was 100% after 2 years and 70% after 5 years with a median OS of 5.75 years. Age (*p = 0.046*), T stage (*p = 0.019*), local control rate (*p = 0.041*) and the time between diagnosis and the first radiation session (*p = 0.01*) had a significant effect on OS. Applied biologically effective dose (BED) did not significantly influence OS or PFS. A 2-year sight preservation rate of 75% could be achieved. In all patients, irradiation could be applied safely without any interruptions due to side effects. Six significant late toxicities with consequential blindness could be observed, making a secondary enucleation necessary in four patients. An impairment of visual acuity due to chronic optic nerve atrophy was identified in five patients within 2 years after treatment.

**Conclusions:**

Linear accelerator-based stereotactic fractionated photon radiotherapy is an effective method in the treatment of uveal melanoma with excellent local control rates and a 2-year vision retention rate comparable to brachytherapy (BRT) or proton beam radiotherapy, even available in small centers and easy to implement. Interdisciplinary decision making is necessary to guarantee best treatment for every patient.

## Background

Uveal melanoma is a rare malignancy with an incidence of six cases per million population in Europe each year, but it is the most common non-cutaneous melanoma [1, 2]. It occurs primarily in elderly men between 60 and 80 years [2]. The role of sunlight and other environmental exposures have been discussed in the literature, but their influence on the pathogenesis is still unknown. For a long time, resection of the tumor with a consecutive enucleation represented the standard of therapy. Over the last four decades RT has become more significant in the treatment of uveal melanoma; not only in the combination with surgery but also as primary treatment. Eye-preserving treatment with RT, especially in the form of brachytherapy (BRT) or proton beam RT, has shown promising results. The Collaborative Ocular Melanoma Study (COMS), a multicenter randomized trial, found no difference in the 5-year overall survival rates of enucleation or BRT in the treatment of small-size or medium-size choroidal melanoma and established the role of BRT in the treatment of uveal melanoma [3–5]. Nevertheless, the visual acuity was impaired in 43% of the cases. Modern RT techniques such as stereotactic RT or proton beam RT promise a better preservation of organs at risk, especially in the therapy of large and peripapillary tumors [6–8].

The aim of this retrospective study is to analyze the value of a fractionated photon RT as eye-preserving treatment and to determine prognostic factors for EFS. Furthermore, negative side effects were compared to other radiotherapy modalities described in the literature. For this purpose, we have systematically analyzed clinical outcomes like enucleation rates due to radiation-induced retinopathy, toxicity and preservation of vision.

## Methods

Patients who underwent treatment with linear accelerator-based stereotactic fractionated and hypofractionated photon RT for uveal melanoma at the Department of Radiation and Oncology of the University Hospital Heidelberg between the years of 1991 to 2015 were included in this analysis; including melanomas of the iris, ciliary body and choroid. Other tumor locations in the orbital cavity as well as other tumor entities were excluded. All patients received primary RT. Two patients were treated with re-irradiation after previous radiotherapy. At the time of the first diagnosis, all patients were examined by an ophthalmologist. Ocular ultrasonography, computed tomography (CT) and magnetic resonance imaging (MRI) were performed to determine tumor stage based on the 2010 American Joint Committee on Cancer (AJCC) TNM (T = tumor, N = lymph node, M = metastasis) classification for uveal melanoma [9] and to exclude distant metastases at the time of the diagnosis. After the treatment, tumor control was assessed every 3 months during the first year, every 6 months during the second year, and subsequently once a year with an ocular examination by the referring ophthalmologist.

Therapy was planned based on a CT scan in irradiation position. For the immobilization of the patients, a scotch-cast mask was used. To determine the target volume, we used a current MRI scan for better demarcation. If a current MRI scan was not available, we performed a CT scan with a contrast agent for matching. Fractionation schedules and prescription doses differed substantially, thus we calculated the biologically equivalent dose (BED) with a tumor α/β of 10 for each patient for better comparability. Radiotherapy was performed in all patients in a stereotactic set-up applying the prescribed dose to the 80% isodose line.

All statistical analyses were performed with IBM SPSS Statistics version 24. Primary endpoints were local progression-free survival (LPFS) and enucleation-free survival (EFS). Secondary endpoints were overall survival (OS) and distant metastasis-free survival (DMFS), acute and late treatment toxicity as well as visual acuity. LPFS was considered to be the time from the last day of treatment to local progression or death from any cause. Local control (LC) was defined as the lack of tumor progression including all cases of stable disease, partial remission and complete remission. Complete remission was defined as a disappearance of the tumor, partial remission as a decrease in the tumor volume > 30% and stable disease when the tumor size did not change after the therapy. EFS was defined as the time between the last day of therapy and enucleation or death from any cause. OS and DMFS were considered as the time period between the first diagnosis and death or appearance of distant metastasis. Survival analysis was performed using the Kaplan-Meier method and the log–rank test.

The log-rank model was used to perform a univariate analysis after determining potentially prognostic factors for survival. WHO (World Health Organization) performance status (0 vs. 1/2), age (median age of 65 years vs. > 65 years), gender (male vs. female), tumor size (T1/2 vs. T3) and treatment dose (median BED ≤100 Gy vs. > 100 Gy) were analyzed for their prognostic significance. A *p*-value ≤0.05 was considered statistically significant. Due to the small number of patients we abstained from a multivariate analysis.

Acute toxicity according to the Common Terminology Criteria for Adverse Events (CTCAE) v4.03 was defined as a toxicity occurring ≤3 months and chronic toxicity > 3 months after the therapy.

## Results

### Patient characteristics

Twenty-four patients were identified and reviewed using the National Center for Tumor Diseases (NCT) cancer registry and the patients’ medical records. In 15 patients the tumor was located in the right eye (62.5%) and in nine patients in the left eye (37.5%). The patients’ characteristics are shown in Table 1. Most patients were initially staged as T2 (*n* = 5; 20.8%) or T3 (*n* = 14; 58.3%), N0 (*n* = 24; 100%) and M0 (*n* = 23; 95.8%). One patient presented with distant metastases at first diagnosis (4.2%). Three patients could not be staged for T stage due to missing data (12.5%). Mean age at the time of the treatment was 63 years (range: 40–89 years). Gender was equally distributed with 50% male and 50% female patients. The majority of the patients had an excellent WHO performance status of 0 (*n* = 14; 58.3%) and 1 at the time of the first examination (*n* = 8; 33.3%).

**Table 1:** Patient characteristics (*n* = 24)

Age		
Median	66 years	
Range	40–89 years	
	n	%
Gender		
Male	12	50.0
Female	12	50.0
WHO Performance Status		
0	14	58.3
1	8	33.3
2	2	8.3
Tumor size		
Tx	3	12.50
T1	2	8.30
T2	5	20.80
T3	14	58.30
Lymph node status		
N0	24	100
N1	0	0
Distant metastases		
M0	23	95.80
M1	1	4.20

### Treatment characteristics

Twenty-two patients underwent primary treatment. The treatment characteristics are depicted in Table 2. In two patients, stereotactic fractionated photon RT was conducted as re-irradiation (8.3%), in one patient after primary proton-beam RT (4.2%), in another patient after primary BRT (4.2%).

**Table 2:** Stereotactic fractionated photon radiotherapy: Results (*n* = 24)

Radiotherapy dose in BED		
Median	78 Gy	
Range	37.5–131.25 Gy	
Follow up	5.2 years	
	n	%
Linear accelerator-based fractionated photon radiotherapy		
Primary irradiation	24	100
Re-irradiation	2	8.3
Local response		
Complete remission	1	4.2
Partial remission	8	33.3
Stable disease	11	45.8
Progressive disease	4	16.7
Enucleation rate	5	20.9
Due to grade 4 toxicity	4	16.7
Due to recurrence	1	4.2
Eye conservation	19	79.1
Blindness before radiotherapy (*n* = 24)	4	16.7
Blindness after 24 months (*n* = 20)		
Due to grade 4 toxicity	5	25.0
Due to recurrence	0	0

The most frequently prescribed hypofractionated dose regimes were 50 Gy in 5 Gy single dose fractions (*n* = 9; 37.5%) and 70 Gy in 7 Gy single dose fractions (*n* = 5; 20.8%). Other dose fractionations were 50 Gy in 2 Gy single dose fractions (*n* = 4; 16.7%), 75 Gy in 7.5 Gy single dose fractions (*n* = 1; 4.2%), 55 Gy in 3 Gy single dose fractions (*n* = 1; 4.2%), 25 Gy in 5 Gy single dose fractions (*n* = 1; 4.2%) and 50 Gy in 10 Gy single dose fractions (*n* = 1; 4.2%). Median total dose was 60 Gy corresponding to a median BED of 78 Gy (range: 37.5–131.25 Gy). Median follow up was 5.2 years.

### Local progression-free survival

**Fig. 1:**
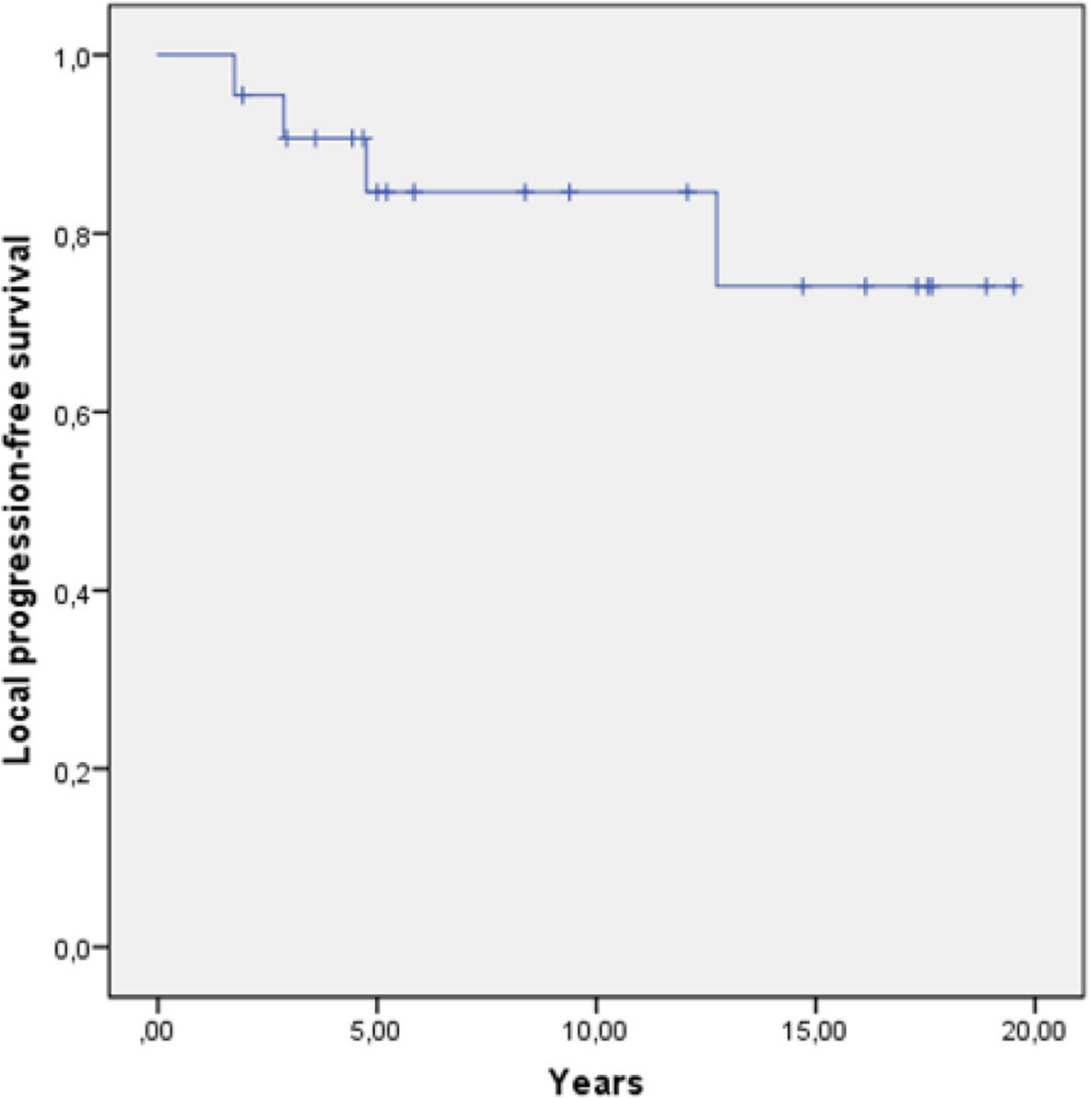
Local progression-free survival in years

**Fig. 2:**
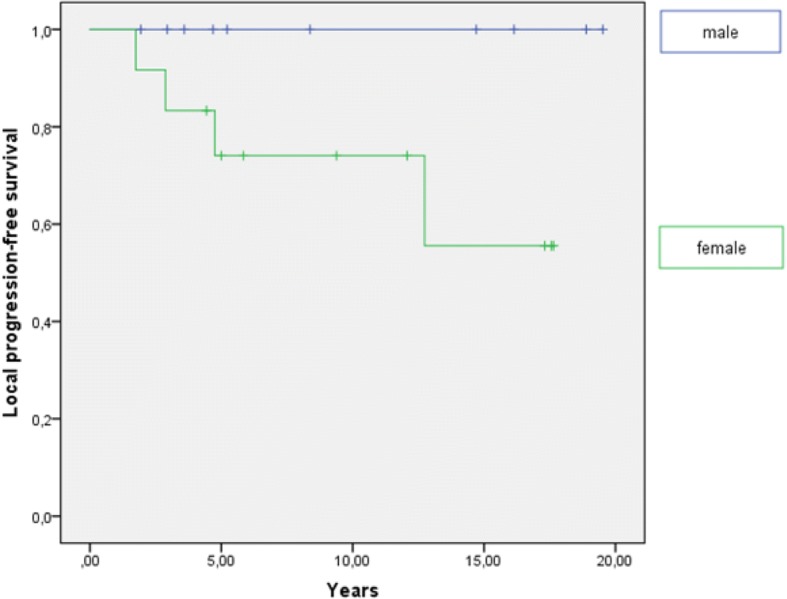
Local progression-free survival dependent on gender. Gender has a significant impact on LPFS (*p* = 0.04)

We observed a good local control rate, with a LPFS of 82% after 5 years (Fig. 1) and a median LPFS of 5.5 years. Gender showed a significant impact on LPFS. We identified a 5-year LPFS of 100% for men and 73% for women (log-rank: *p = 0.04*; Fig. 2). Hence, all local progressions occurred in women (*n* = 4; 100%). Enucleation rate (*p = 0.804*), age (*p = 0.472*), T stage (*p = 0.517*), time period between the first diagnosis and the beginning of radiotherapy (*p = 0.535*), BED (*p = 0.839*) as well as performance status (*p = 0.85*) were not identified as prognostic factors for LPFS in the univariate analysis. At the time of last assessment, complete response could be observed in one patient (4.2%), partial response in eight patients (33.3%), stable disease in 11 patients (45.8%) and progressive disease in four patients (16.7%). The overall response rate including complete response, partial response and stable disease was 86.4% (*n* = 20). Three of four progressions occurred within the first 5 years after therapy. All progressions were diagnosed within the radiation field. Two patients with locally progressive disease were treated with re-irradiation, one patient with enucleation and another patient with eye-preserving salvage surgery.

#### Enucleation-free survival

**Fig. 3:**
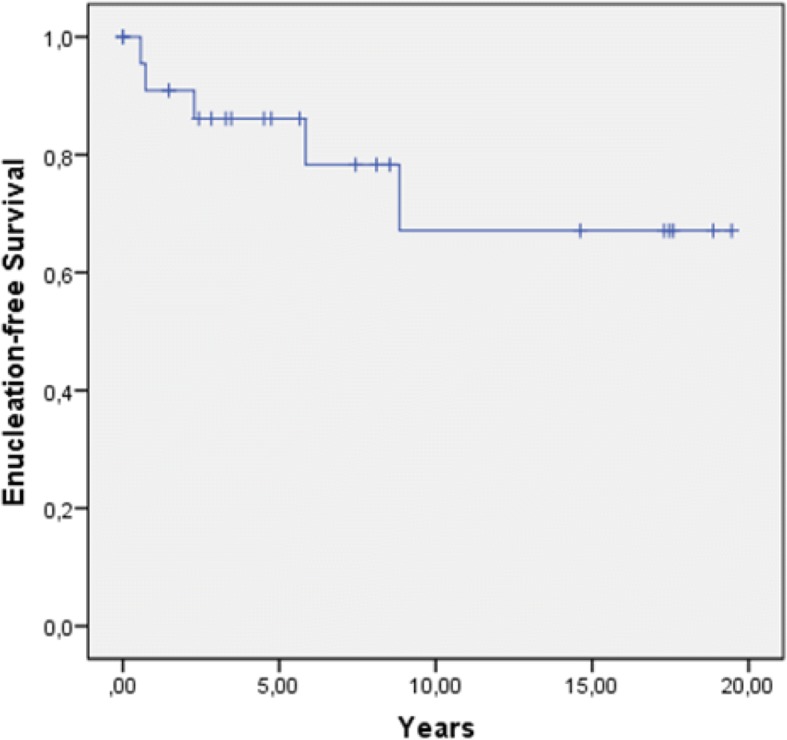
Enucleation-free survival in years

**Fig. 4:**
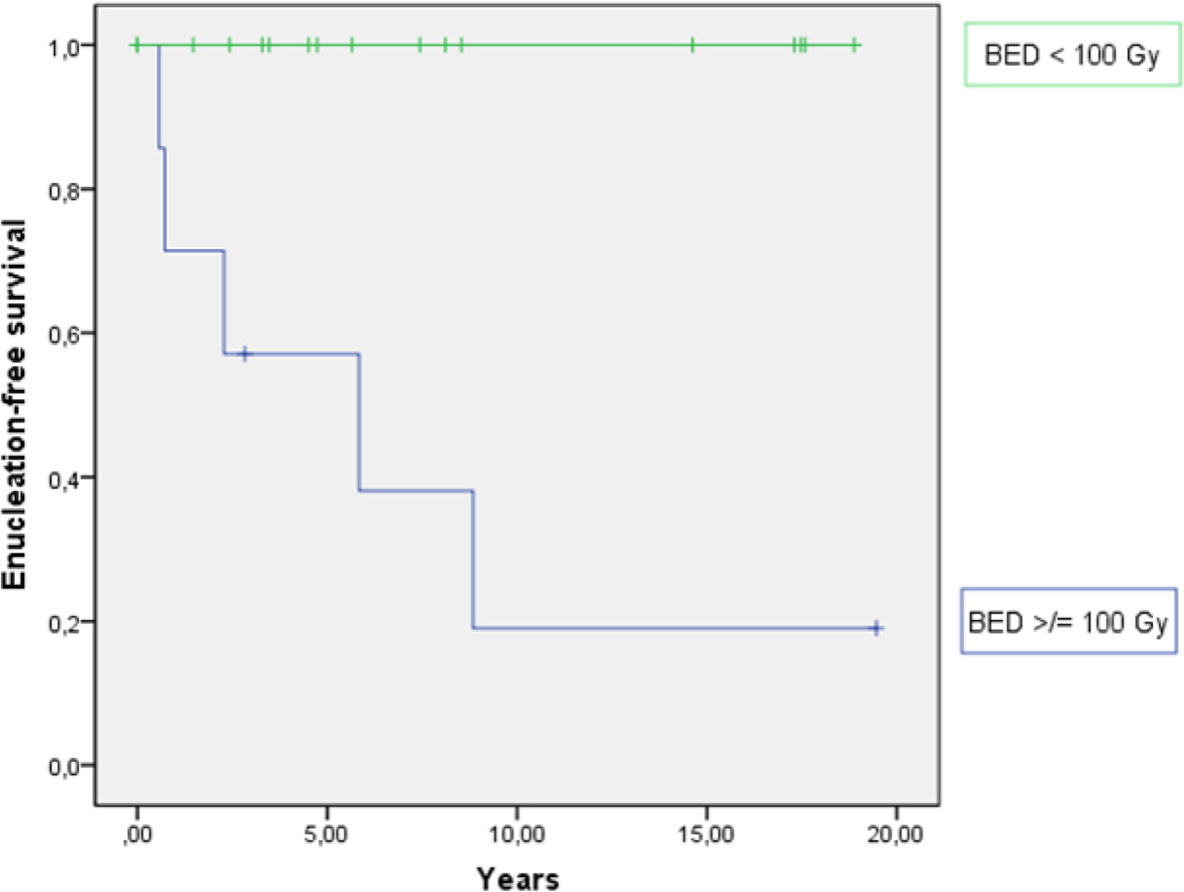
Enucleation-free survival dependent on the BED. A BED ≥ 100 Gy has a significant worse impact on EFS (*p* < 0.0001)

**Fig. 5:**
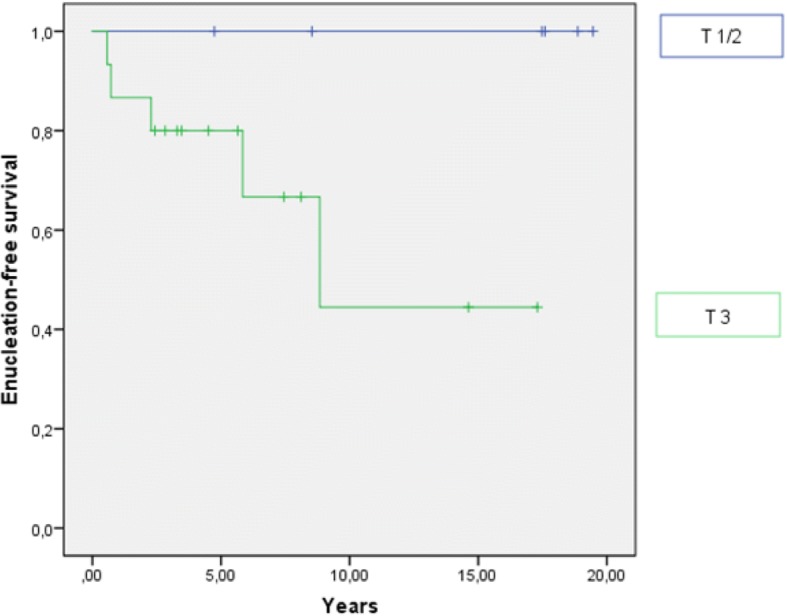
Enucleation-free survival dependent on T stage. T3 stage has a negative impact on EFS (*p* = 0.069)

During follow-up, five patients underwent enucleation and four of them were women (80.0%). The overall enucleation rate was 21% with a 2-year and 5-year EFS of 88 and 83%, respectively (Fig. 3). All enucleations occurred in patients who were irradiated with a BED ≥100 Gy and had a higher T stage (T1/2 vs. 3). In the univariate analysis, we established a BED ≥100 Gy as a prognostic factor for worse EFS (*p* < 0.0001; Fig. 4). T stage showed no significant impact on EFS (*p* = 0.069), but the curve progression suggests a negative influence of T3 stage on the EFS (Fig. 5). Indeed, tumor size correlated significantly with the enucleation rate (log-rank: *p* = 0.01). Importantly, there was no correlation between local recurrence and EFS (*p* = 0.823). Data of histopathological analysis of secondary enucleated eyes were available in three of five cases. Only one enucleation was due to a locally recurrent disease (4.2%) with 35 mitotic figures per 50 HPF identified. In fact, most enucleations were conducted due to side effects of radiotherapy (*n* = 4; 80%). In two cases, pronounced necrosis could be identified histopathologically.

### Overall and distant metastasis-free survival

Median overall survival (OS) was 5.75 years for all patients with a 2-year and 5-year OS rate of 100 and 70%, respectively. Univariate analysis for prognostic factors showed a significant impact of age (*p = 0.046*), local progression rate (*p = 0.041*), T-stage (*p = 0.019*) and time between the initial diagnosis and the beginning of the therapy (*p = 0.01*) on OS. A significantly lower 2-year OS could be registered in patients who were older than 65 years (60% vs. 88%), had a T3 tumor (66% vs. 83% for T1/2) or a local progression (50% vs. 76%) and who did not started the treatment within 4 weeks after the initial diagnosis (44% vs. 64%). Gender (*p = 0.73*), performance status (*p = 0.718*), BED (*p = 0.478*) and the distant progression rate (*p = 0.103*) did not correlate with OS.

Distant metastases developed in seven patients (29%), five within the first 5 years after the initial diagnosis (20.8%). Metastases involved the liver in one case (4%), the brain in one case (4%), the liver and the lungs in three cases (13%), the liver, the lungs and the brain in one case (4%) and cervical lymph nodes in one case (4%). Six of seven patients received palliative systemic therapy including Sorafenib (*n* = 1, 4%), Interferon (*n* = 1, 4%), Gemcitabine (*n* = 1, 4%), Gemcitabine/Treosulfam (*n* = 1, 4%) and Dacarbazin, Vindesin and Cisplatin (*n* = 2, 8%). All patients with metastatic disease and palliative systemic therapy died after a median time of 12 months after first diagnosis of the metastases. One patient received systemic unilateral neck dissection for lymph node metastases and was still alive at last follow up. 2-year and 5-year DMFS were 90 and 59%, respectively. In the univariate analysis, we identified a statistically significant negative impact of T3 stage (*p* = 0.*027*), local progression rate (*p = 0.038*) and time between initial diagnosis and beginning of radiotherapy > 4 weeks (*p = 0.004*) on DMFS. Performance status (*p = 0.850*), gender (*p = 0.670*), age (*p = 0.076*) and BED (*p = 0.526*) did not statistically significantly correlate with DMFS.

### Toxicity and visual acuity

Acute toxicity during and 3 months after radiotherapy was assessed in all patients (*n* = 24). The therapy was tolerated well with no grade 4 acute toxicities according to CTCAE v4.03. Thirteen patients did not develop any acute side effects (54%). Among all patients, mild unilateral conjunctivitis occurred in seven patients (29%) and was the most frequent acute toxicity. Skin reaction with erythema and edema was recorded in four patients (17%), of whom two patients had a grade 1 (8.3%), one patient a grade 2 (4.2%) and another patient a grade 3 acute skin reaction (4.2%).

We had to exclude three patients from the analysis of late toxicities because of incomplete medical records (12.5%). Chronic radiogenic side effects occurred in 12 patients (57.1%); in six cases within the first year (28.6%) and in two cases within 2 years after therapy (9.5%). Long term adverse side effects occurred in four patients (19.0%). Within the first 6 months after therapy, we identified a grade 1 macula edema in two patients (9.5%), and slight sicca symptoms in another patient (4.7%). One year after therapy, one patient developed a grade 2 toxicity with cloudiness of the lens and a slight visual restriction (4.7%). A grade 3 ischemic retinopathy with vision restriction occurred in two patients after two and 12 years (9.5%), a grade 3 glaucoma in one patient 8 years after therapy (4.7%). During follow up, six patients suffered from a grade 4 retinopathy with consequential blindness (25%). Four patients received an enucleation due to central artery obliteration, retinal detachment, recurrent painful corneal ulcer or vitreous hemorrhage after a mean time of 2 years after irradiation (19.0%). Two patients went blind because of retinal detachment without enucleation, both nearly 1 year post therapy (9.5%).

We measured optic nerve damage indirectly with the preservation of sight within 24 months after therapy. We excluded ten patients from the analysis (41.7%) who were initially blind (*n* = 4; 16.7%), became blind because of other treatment-related side effects (*n* = 5; 20.8%) or had a tumor recurrence in this time period (*n* = 1; 4.2%). Initially, four patients suffered from tumor caused blindness (16.7%) and 20 patients had visus restrictions (83.3%). Visus was measured by an ophthalmologist at the beginning of the therapy and 3 months, 6 months, 12 months and 24 months after therapy. We systematically analyzed the records of the remaining 14 patients (62.5%). We defined a visus loss over 1/3 compared to the initially measured visus as visual impairment, a visus increase over 1/3 as improved visual acuity and all other results as stable visus. Six weeks after therapy, 11 patients had a stable visus (73.3%), two patients showed an improvement of the initial visus (13.3%) and one patient had a visual impairment (6.7%). Within 6 and 12 months, two more patients developed a visual impairment (overall *n* = 3; 20%). Five patients with visual impairment (35.7%), four patients with stable visus (28.6%) and five patients with visual acuity (35.7%) could be identified within 24 months after therapy. No blindness due to optic nerve atrophy occurred in this time period. Therefore, we identified a 2-year sight-conservation rate of 75%.

## Discussion

In the 1970s, enucleation was considered as the only therapy option in the treatment of uveal melanomas. In 1998, the COMS, a prospective trial, compared two therapy modalities, enucleation and BRT. Large tumors with more than 18 mm in diameter and 8 mm in height were excluded from the analyses. The authors could demonstrate an equal 5-year survival rate of 81% for both therapy options. Thus, BRT was established as an eye-preserving treatment for small and medium sized uveal melanomas [3–5]. Nowadays, the therapy standard consists of plaque brachytherapy and charged particle therapy with protons [10, 11]. Additionally, other treatment modalities like stereotactic radiosurgery with gammaknife or cyberknife as well as stereotactic fractionated photon radiotherapy gain in importance [6, 12–14]. Prospective studies comparing external beam RT and BRT are still missing. In the current study, we analyzed 24 patients treated with stereotactic photon beam RT. In contrast to the COMS, we treated a great number of large sized tumors. However, we assessed adequate survival rates with a five-year OS, LRFS and DMFS of 70, 82 and 59%, respectively. Although applied doses vary widely for SRS /FSRT, 5-year OS between 60 and 90%, LC between 87 and 97% as well as eye retention rates between 77 and 90% are described in the current literature [14–16].

Nowadays, enucleation is only used in recurrent disease of medium sized melanomas or in large tumors without the option of other eye-preserving therapies. In our analyses, a 5-year eye-preservation was achieved in 83% and a 2-year preservation of the visual acuity in 75% of the patients. The overall enucleation rate was 20.8%. In the current literature, the eye preservation rate fluctuates between 72 and 97% after BRT and between 76 and 89% for charged particles [17–24]. Verschueren et al. identified an eye-preservation rate of 96% for BRT in the treatment of small and intermediate sized tumors [20]. In their analyses, visual acuity was preserved in 52%. In a comparable trial by Yazici et al. the authors identified a 5-year eye-preservation rate of 73% for LINAC based SRS/FSRT with 1–3 fractions conducted via cyberknife [12]. Furdova et al. could even show an eye-retention rate of 88% after one step LINAC based SRS with a single dose fraction of 35 Gy for T2 or T3 uveal melanoma [25]. Generally, outcome of long term visual acuity is poor after RT. Gragoudas et al. reported a decreased visual acuity < 0.1 Snellen 5 years after proton beam RT [26]. Modorati et al. described a high rate of visual loss after follow up for gamma knife radiosurgery [15]. During the first 2 years after RT, visual acuity decreased in none of our patients. Stereotactic photon RT in the treatment of large uveal melanomas seems to be an effective method for eye and visual preservation.

Furthermore, we identified prognostic factors for OS, LPFS, DMFS and EFS. Gender had a significant impact on LPFS with higher tumor progression rates for women. T stage was the strongest prognostic factor in the univariate analysis and had an impact on the OS, DMFS and the EFS. Although tumor size could not be identified as independent prognostic factor on EFS, we could show a significant impact of T stage on the enucleation rate, possibly due to the small number of patients analyzed in the current study. In accordance, Bensoussan et al. described a five-year OS for T3 stage vs. T4 stage of 68% vs. 52% [27]. Several other authors reported higher tumor size resulting in higher enucleation rate [23, 28–30]. Furthermore, we showed a negative influence of a BED > 100 Gy on the EFS especially due to negative side effects of the therapy. Whether higher enucleation rate is directly linked to higher prescribed doses or depends more on higher T stage as well as larger RT field with consequently higher doses to the critical organs still remains unclear.

Nevertheless, it is known that nearly 20% of all irradiated patients suffer under enucleation after therapy and that a considerable proportion is caused by side effects. For good local control, high doses are necessary. Furdova et al. reported a 5-year local control rate of 85% while secondary enucleation due to complications like treatment-related neuropthy and secondary glaucoma was necessary in 12% of the patients after single dose SRS with 35 Gy [25]. Dieckmann et al. reported a good local control rate of 98% and enucleation rate of 7.7% when a dose between 50 Gy and 60 Gy in 5 Gy single dose was applied according a BED between 75 Gy and 90 Gy. Nevertheless, retinopathy, cataract and optic nerve damage occurred in 25.5, 18.9% and in 20% of the patients [13]. Other studies described similar results in the local control rate with 2-year and 5-year local control rates of 100 and 96% and no enucleations when only 50 Gy was applied on the tumor in 5 Gy single dose fractions corresponding a BED of 75 Gy [31]. Dose optimization is necessary for achieving good local control rates and adequate preservation of the organs at risk to provide high enucleation rates. Gragoudas et al. could not show a benefit for visual acuity when radiotherapy dose was reduced from 70 Gy to 50 Gy for protons [32].

In the current analysis 80% of the enucleations (overall 20.8%) were caused by grade 4 toxicities. Bensoussan et al. reported a similar enucleation rate of 19.5% for large tumors which were treated with BRT, the majority due to negative side effects [27]. In the current study 2-year and the 5-year chronic toxicity rates of 38 and 57% could be identified. A meta-analysis by Wang et al. showed equal enucleation rates and survival rates for charged particle radiotherapy in the treatment of uveal melanomas compared with stereotactic photon radiotherapy and BRT [10]. The authors found a significantly lower toxicity rate of cataract formation and radiation-induced retinopathy. Thus, particle therapy is used in large melanomas with paripapillary location for better organ at risk conservation. However, in recent years, hypofractionated stereotactic radiotherapy has been shown to be an effective alternative to protons [13, 31, 33]. Although data with larger case series are available nowadays, e.g. by Yazici et al. and by van den Bosch et al., prospective studies are still lacking [12, 28]. Nevertheless, the current data situation regarding the effect of FSRT on EFS with adequate follow-up times to assess long-time effects of therapy is thin. Therefore, we think that the current study could strengthen prior results despite its retrospective character, small number of patients and various RT schedules applied.

## Conclusions

Stereotactic hypofractionated RT is a safe alternative to surgical eye enucleation in uveal melanoma patients, yielding good local control rates as well as allowing eyesight preservation and avoidance of surgery in the majority of patients.

## Abbreviations

AJCC: American Joint Committee of Cancer
BED: Biological effective dose
COMS: Collaborative Ocular Melanoma Study
CT: Computed tomography
CTCAE: Common Terminology Criteria for Adverse Events
DMFS: Distant metastases-free survival
EFS: Enucleation-free survival
LC: Local control
LPFS: Local progression-free survival
MRI: Magnetic resonance imaging
NCT: National center for tumor diseases
OS: Overall survival
RT: Radiotherapy
TNM: Tumor, lymph node, metastasis
